# Supplementary material for: Hepatocyte Nuclear Factor 4α (HNF4α) Plays a Controlling Role in Expression of the Retinoic Acid Receptor β (RARβ) Gene in Hepatocytes
Source: Int J Mol Sci. 2023 May 11;24(10):8608. doi: 10.3390/ijms24108608 (PMC10218549; doi:10.3390/ijms24108608)
Supplement: Supplementary file 1 [file ijms-24-08608-s001.zip › ijms-2352806-supplementary.pdf]

Supplementary Materials

Table S1. Primers used for mouse liver genes.

Gene name	Sense primer	Antisense primer
RBP4	5′-ACTGGGGTGTAGCCTCCTTT-3′	5′-ACTGCCTCTCTAGGCACAGC-3′
PERP	5′-TCATCCTGTGCATCTGCTTC-3′	5′-GGGTTATCGTGAAGCCTGAA-3′
Saa4	5′-TTGCTCGTGATTCACTCCTG-3′	5′-TCTCCACAAGTCCCAAGTCC-3′
Rdh5	5′-CCACACCATGGCTAACACAG-3′	5′-CTTCAGGGTGCTCTCCAGAC-3′
Rdhe2	5′-ATCAGTTAGCTCGGGAAGCA-3′	5′-ATGAGGTCATCTGGGCAGTC-3′
Rarres2	5′-GTGCACAATCAAACCAAACG-3′	5′-GGCAAACTGTCCAGGTAGGA-3′

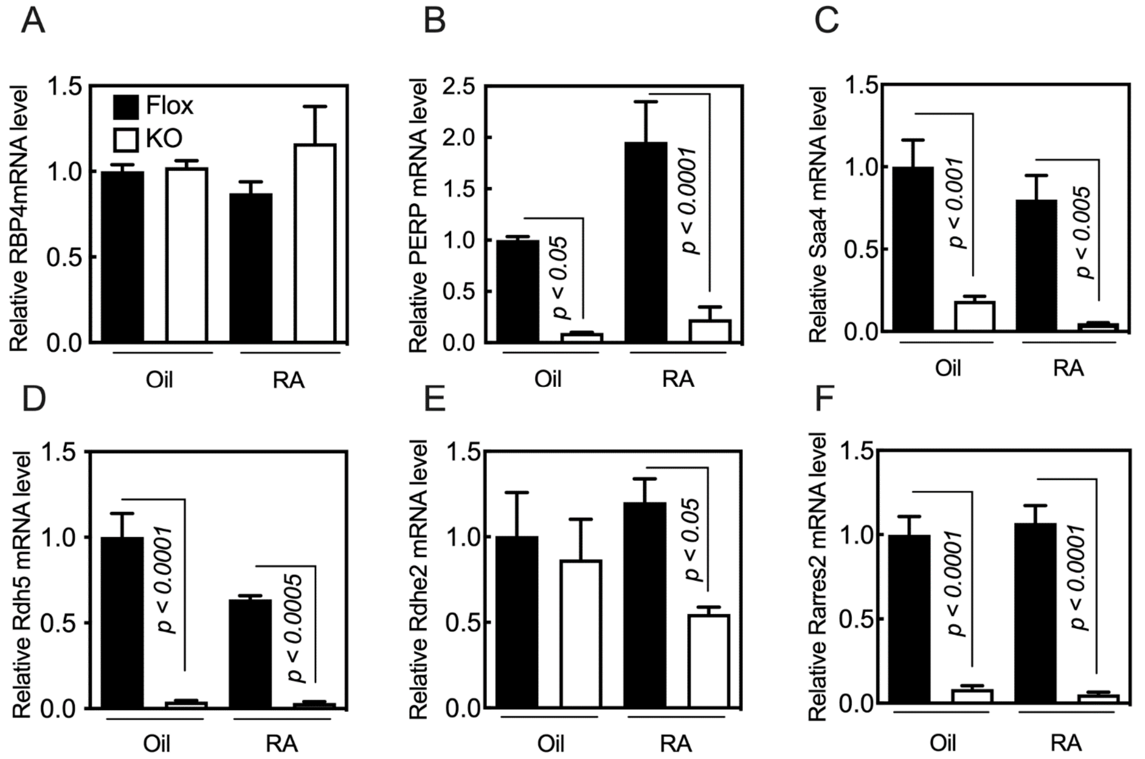

Figure S1. Expression of a few genes in the liver were examined to check the integrity of the *Hnf4a*$^{F/F;AlbERT2cre}$ mouse line as the controls. *Hnf4a*$^{F/F}$ (Flox) and *Hnf4a*$^{F/F;AlbERT2cre}$ (KO) mice were administered either oil as the vehicle control (0.1 ml/ mouse) or RA in oil (0.25 mg/ml, 0.1 ml/ mouse) for 6 h and then sacrificed for collection of liver tissue samples. Total RNA was extracted from tissue samples for qPCR analysis of gene expression using gene specific primers (Table S1).
